# Stress-Easing Effect of Diacyl Glyceryl Ethers on Anxiety-Related Behavior in Mice

**DOI:** 10.3390/foods13233765

**Published:** 2024-11-24

**Authors:** Rong Jiang, Takeshi Ohkubo, Toshihiko Sato, Nobuyuki Sakai

**Affiliations:** 1 Department of Psychology, Graduate School of Arts and Letters, Tohoku University, Kawauchi 27-1, Aoba-ku, Sendai 980-8576, Miyagi, Japan; jiang.rong.p8@dc.tohoku.ac.jp; 2 Faculty of Human Sciences, Department of Health and Nutrition, Sendai Shirayuri Women’s College, Honda-cho 6-1, Izumi-ku, Sendai 981-3107, Miyagi, Japan; t-ohkubo@sendai-shirayuri.ac.jp; 3 Department of Psychology and Humanities, College of Sociology, Edogawa University, Komagi 474, Nagareyama 270-0198, Chiba, Japan; toshi-s@edogawa-u.ac.jp

**Keywords:** stress, anxiety-related behavior, mice, diacyl glyceryl ethers, functional foods

## Abstract

Stress and anxiety are significant psychological challenges in modern society, which have led to a rapidly growing market for functional foods, including those reported to relieve stress, as alternatives to psychoactive drugs. Among these, diacyl glyceryl ethers (DAGE) derived from deep-sea shark liver oil have gained attention for their strong antioxidant properties and potential mental health benefits. Building on preliminary evidence suggesting DAGE’s efficacy in enhancing stress resilience and modulating biochemical pathways associated with reduced oxidative stress, the present study aimed to examine their effects on stress responses in two specific mouse strains. Each mouse was fed either a DAGE-infused diet or a control diet for three weeks. Their stress responses were evaluated using three behavioral tests: the elevated plus maze, open-field, and forced swimming tests. The DAGE-fed mice displayed lower stress responses than the control mice in the initial trial of each test. Specifically, DAGE-fed mice demonstrated increased time spent in the open arms in the elevated plus maze and more time spent in the center of the open field, suggesting reduced anxiety. Additionally, in the forced swimming test, DAGE-treated mice displayed reduced immobility times, indicating potential antidepressant effects on the mice. These findings suggest the potential of DAGE to bolster stress resilience in mice, emphasizing their promise for further studies in human stress management.

## 1. Introduction

In contemporary society, the use of nutritional supplements to enhance quality of life is gaining popularity. These supplements are often associated with significant health benefits [1,2,3,4,5,6]. For instance, research involving mice suggests that prebiotics can bolster resistance to depression and mitigate the effects of chronic stress [7]. Historically, polyunsaturated fatty acids obtained from sources such as fish and sesame oils have been used for alleviating pain and addressing cardiovascular issues [2,3,5,8]. Furthermore, combinations of shark and sesame oils have shown promise in reducing depression in animal studies, indicating their potential as effective supplements [8]. Notably, eicosapentaenoic acid (EPA) and docosahexaenoic acid (DHA) derived from salmon oil are well-regarded for their roles in stress and anxiety management [1,2,9].

Recently, diacyl glyceryl ethers (DAGE) extracted from deep-sea shark liver oil have been recognized for improving sleep quality and stress responses in humans. DAGE play a crucial role in the body’s antioxidant defenses by serving as a precursor to plasmalogens [10]. These compounds help combat oxidative stress in the brain, thereby safeguarding the nervous system [11]. Studies indicate that sleep deprivation can reduce plasmalogen levels, negatively affecting cognitive and emotional health [12]. Thus, increasing DAGE intake could enhance the brain’s resilience to oxidative stress and support mental well-being by maintaining cognitive function and mood stability [13]. Human perception of stress can be influenced by numerous factors, making it a complex phenomenon to study. This variability underscores the importance of using animal models, such as those involving mice, in research. Mice provide a controlled, consistent environment for examining the mechanisms through which DAGE influence anxiety-related behaviors and contribute to stress management. This study aimed to deepen our understanding of the psychological benefits of DAGE, with the goal of potentially guiding future dietary recommendations for mental health optimization [14].

We conducted three behavioral experiments, including the elevated plus maze (EPM) test [15,16], open-field test (OFT) [17,18], and forced swimming test (FST) [19,20,21], to evaluate the potential effects of DAGE on anxiety-related behavior in two mouse strains, BALB/cCrSIc and C57BL/6NCrSIc, which are known for their differing stress response strategies. The EPM test examined the ameliorative effects of DAGE on the exploratory behavior of mice in novel environments and their anxiety stemming from conflict behaviors due to the elevated open arms. The OFT was designed to measure whether DAGE ameliorate general anxiety behavior in mice. The FST was designed to investigate the antidepressant effects of DAGE in mice. Additionally, our research sought to explore behavioral differences between two testing sessions across these experiments [16,22,23,24,25,26,27,28,29]. By using the BALB/c and C57BL/6 strains, both of which are commonly used in anxiety behavior studies [30], our goal was to deepen our understanding of the capacity of DAGE to mitigate various stress forms. Our objective was not only to advance the studies using animal models but also to uncover evidence with significant implications for enhancing human mental health.

## 2. Materials and Methods

### 2.1. Diacyl Glyceryl Ethers

Diacetyl glyceryl ethers (DAGE), derived from deep-sea shark liver oil, has garnered interest as a functional food with potential anxiolytic and antidepressant properties, attributed to its strong anti-inflammatory and antioxidant capabilities [11,12,13]. The diets for the present study, including the DAGE-enriched and control chow, were custom-made (see Table 1 for details). The concentration of DAGE was established at the highest safe intake level, as determined by a two-week toxicity study in mice. We selected a single dose of DAGE based on specific safety and efficacy outcomes reported in the prior study [13], which demonstrated that a particular dose (360 mg/day) improved REM sleep quality and stress responses in humans. Adjusting for body weight differences between humans and mice, we administered DAGE at 1.5% (*w*/*w*) of the feed. This dosage aligns with the effective levels established in the prior research [13] and was confirmed to be safe in prior toxicological evaluations (unpublished results from Bozo Research Center Inc. Tokyo, Japan), showing no adverse health effects even at higher concentrations. The raw material for DAGE was sourced from a commercial deep-sea shark liver oil product provided by Maruha Nichiro Co., Ltd (Tokyo, Japan).

### 2.2. Experimental Animals

Forty-eight male mice, specifically 24 BALB/cCrSIc and 24 C57BL/6NCrSIc, each six weeks old and weighing between 19–24 g, were sourced from Kumagai-shigeyasu Co., Ltd., Sendai, Japan. The experimental protocols involving these mice adhered to the guidelines for experimental animals and received approval from the Ethics Committee of the Center for Laboratory Animal Research at Tohoku University, Japan (Approval no. 2020BUNDOU-001). The mice were housed under standard conditions free from specific pathogens, with a temperature maintained at 23 ± 2 °C and a 12:12 h light/dark cycle. Prior to the experiments, all mice underwent a three-week adaptation period where they were fed commercial chow provided by Oriental Yeast Co., Ltd.(Tokyo, Japan). Following this, they were randomized and assigned to various groups (N = 24/group) as outlined in Table 2.

### 2.3. Behavioral Assessments

#### 2.3.1. Elevated Plus Maze Test

In this study, we assessed the anxiolytic effects using the EPM test, a widely validated method for evaluating anxiety-related behaviors [15,16,31]. The EPM, known for its high sensitivity, effectively measures the impact of both anxiolytic and anxiogenic drugs [32]. The apparatus, supplied by O’HARA & CO., Ltd. (Tokyo, Japan), features two open arms (25 × 5 × 0.5 cm) intersecting with two enclosed arms, which have 16 cm high walls, and a central platform (5 × 5 × 0.5 cm). The open arms are bordered by minimal walls (0.5 cm high) to prevent the mice from falling. Positioned 50 cm above the ground and within a protective enclosure, the maze ensures safety during the tests. Testing began by placing a mouse at the maze’s center, facing a closed arm, allowing free movement [15]. Each session lasted 10 min and was documented via a video camera. Post-test, the apparatus was meticulously cleaned: first wiping away any waste, then washing with tap water, followed by disinfection with super hypochlorite solution, and a final wipe to remove olfactory traces. Behavioral data recorded included the latency to entering the open arms, total distance traveled, and the percentage of time spent in the open arms (PTOA), which served as indicators of anxiolytic activity.

#### 2.3.2. Open-Field Test

The OFT is a widely recognized model for assessing anxiety-related behaviors in animals and is appreciated for its straightforward methodology and the reliability of its data [17,18]. In this study, the OFT was employed to examine whether DAGE could mitigate anxiety in mice introduced to a new environment. The OFT apparatus consisted of a circular polyvinyl chloride washtub measuring 35 cm in diameter and 13.5 cm in height, with a central area diameter of 17.5 cm. During the test, mice were individually placed at the center of the apparatus and allowed to explore for 10 min. We recorded the percentage of time spent in the central area, the number of entries into the central area, and the total distance traveled as measures of anxiety. Following each session, the apparatus was cleaned using the same procedure as described for the EPM test, ensuring the removal of any residues and olfactory cues.

#### 2.3.3. Forced Swimming Test

The FST is a well-established method for evaluating potential antidepressant effects in rodent models. In this test, rodents are placed in a water tank to measure their escape-driven movements, which are carefully quantified [19,20,21]. This experiment aimed to determine if the continuous intake of DAGE enhances antidepressant-like behaviors in mice.

For the FST, we utilized a transparent Plexiglas columnar tank measuring 55 cm in height and 22 cm in diameter. The water was maintained at a level of 15 cm from the bottom, with a temperature kept between 23 and 25 °C. Mice were gently lowered into the water by the tail to avoid submerging their heads, and they were allowed to float for a duration of 6 min [19]. The test concluded if the mice began diving or could no longer maintain a floating posture.

The primary focus of the behavioral analysis in the FST was to accurately measure the duration of immobility, which is indicative of depressive behavior. Immobile behavior is defined as the minimum movement necessary to keep the head above water and stabilize the body [19]. Additionally, a state of floating characterized by minimal motion following a period of active movement was also considered an indication of immobility, representing a cessation of effort due to expended kinetic energy.

### 2.4. General Protocol

**Figure 1:**
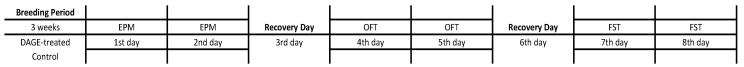
Behavioral experiment procedure. EPM, elevated plus maze; OFT, open-field test; FST, forced swimming test.

The animals were divided into DAGE-treated and control groups, receiving their respective substances for three weeks. The anxiolytic and antidepressant effects were evaluated using the EPM test, OFT, and FST. Over an 8-day behavioral testing period, all mice underwent these experiments sequentially, starting with the EPM test, followed by the OFT, and concluding with the FST, as illustrated in Figure 1.

The OFT was conducted one day after the EPM test, based on evidence from previous studies [9,33] indicating that this sequence does not influence the outcomes of the OFT. The FST, being the most stressful of the three tests, was conducted last to avoid any potential impact on the results of the EPM test and OFT.

### 2.5. Statistical Analysis

#### 2.5.1. Elevated Plus Maze

Behavioral data were collected and analyzed using MouBeAT, developed by Elísabet Bello-Arroyo at the Centro Nacional de Investigaciones Cardiovasculares Carlos III, Madrid, Spain, based on the public domain Image J program [34]. Behavioral data were analyzed using two groups (control group vs. DAGE-treated) × two test days (1st day vs. 2nd day), a two-way repeated measure of analysis of variance (ANOVA), followed by post hoc analysis (Turkey). Probability values less than 0.05 (*p* < 0.05) were considered statistically significant.

#### 2.5.2. Open-Field Test

Behavioral data were collected and analyzed using MouBeAT [34]. In each strain of mice in the two groups during the two test days, behavioral data were analyzed using two-way (2 Groups × 2 Test days) repeated measures of ANOVA, followed by post hoc analysis (Turkey). Probability values less than 0.05 (*p* < 0.05) were considered statistically significant.

#### 2.5.3. Forced Swimming Test

The experimental protocol for the FST typically lasts 6 min from start to end. However, in general, only the last 4 min of the test were analyzed, as mice display high levels of activity during the initial 2 min, which may mask the effects of the treatment [19].

In the FST, for each strain of mice in the two groups during the two test days, the immobility time was analyzed using two-way (2 Groups × 2 Test days) repeated measures of ANOVA, followed by post hoc analysis (Turkey). Probability values less than 0.05 (*p* < 0.05) were considered statistically significant.

#### 2.5.4. Changes in Body Weight per Food Intake

Several studies have shown that stress can either increase or decrease food intake in both rodents and humans [35,36,37]. In order to further investigate whether DAGE alleviates stress responses, such as stress-induced feeding deficits, following the three behavioral experiments, we conducted an unpaired *t*-test on the food intake (food consumption relative to body weight) within 24 h following each test. Probability values less than 0.05 (*p* < 0.05) were considered statistically significant. It is important to note that following the second session of the FST, all mice were humanely euthanized. Consequently, data on food intake post-FST are only available for the 24 h period following the first session of this test. This measure acts as a physiological reference to further clarify the relationship between stress relief and DAGE treatment.

## 3. Results

### 3.1. Behavioral Assessments

#### 3.1.1. Elevated Plus Maze Test

**Figure 2:**
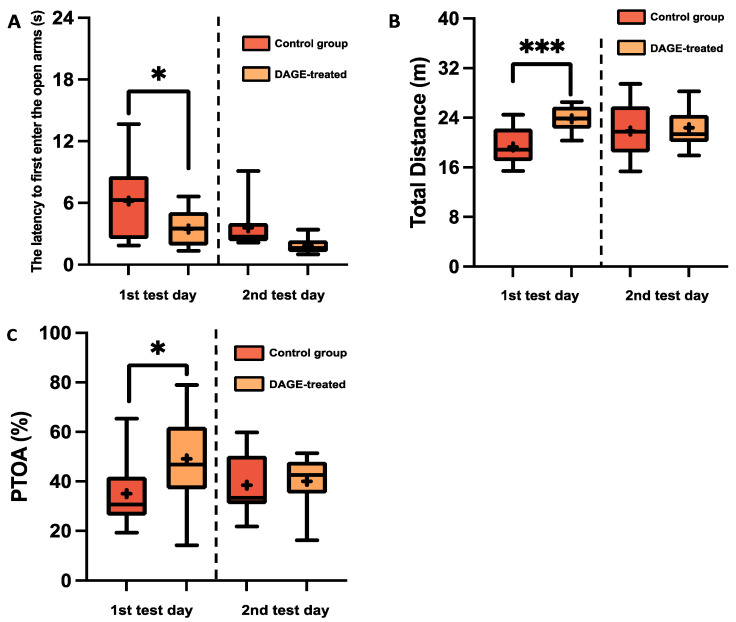
Box-and-Whisker Plots of Behavioral Metrics in BALB/c Mice: The plots illustrate the distribution of the following: (**A**) Latency to first entering the open arms; (**B**) Total distance traveled; (**C**) Percentage of time spent in open arms for DAGE-treated and control (non-DAGE-treated) groups. The boxes represent the 25th and 75th percentiles, while the whiskers extend to the 5th and 95th percentiles. The black lines within the boxes denote median values, and the “+” symbols indicate mean values. Asterisks indicate significance levels, with * and *** representing *p*-values less than 0.05 and 0.001, respectively.

The results of the anxiolytic effects of DAGE in the BALB/c strain, evaluated through the EPM test, are presented in Figure 2A–C. The data from the first test day showed a significant difference between the DAGE-treated mice and the control group. Specifically, DAGE-treated mice exhibited a significantly shorter latency to first entering the open arms (*p* < 0.05), as shown in Figure 2A. Additionally, the total distance traveled by DAGE-treated mice was significantly greater (*p* < 0.001) than that of the control mice on the first test day (Figure 2B). Moreover, PTOA was significantly longer for the DAGE-treated mice compared to the control group on the same day (*p* < 0.05), indicating a reduced anxiety level, as depicted in Figure 2C.

**Figure 3:**
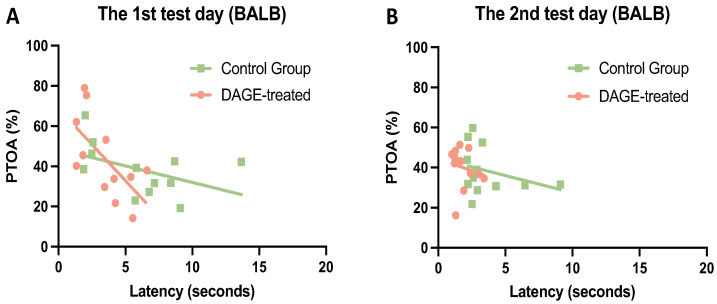
Correlation Between DAGE Treatment and Relief of Anxiety-Related Behavior: The latency to the open-arm end is used to present exploratory behavior. The PTOA is used to denote an anxiety state. (**A**) Shows a linear relationship between PTOA and latency to the open-arm end in DAGE-treated mice (orange symbols and line) as well as between PTOA and latency to the open-arm end in control mice (green symbols and line), indicating the correlation between the effect of DAGE-treatment and the relief of anxiety-related behavior on the first test day. (**B**) Demonstrates that on the second test day, the correlation was not observed (DAGE-treated, *R*^2^ = 0.092, *p* = 0.336; Control, *R*^2^ = 0.033, *p* = 0.572).

We further analyzed the relationship between the effect of DAGE and anxiety-related behavior in the DAGE-treated and control mice. The PTOA was used as an index of anxiety relief. Figure 3A displays negative correlations between PTOA and latency to entering the open arms among DAGE-treated mice (orange symbols in Figure 3A, *R*^2^ = 0.405, *p* < 0.05), which was not observed in the control group (green symbols in Figure 3A; *R*^2^ = 0.208, *p* = 0.136) on the first test day. These correlation curves and their shifting toward higher levels after DAGE treatment suggest the role of DAGE in relieving anxiety-related behaviors on the first test day.

**Figure 4:**
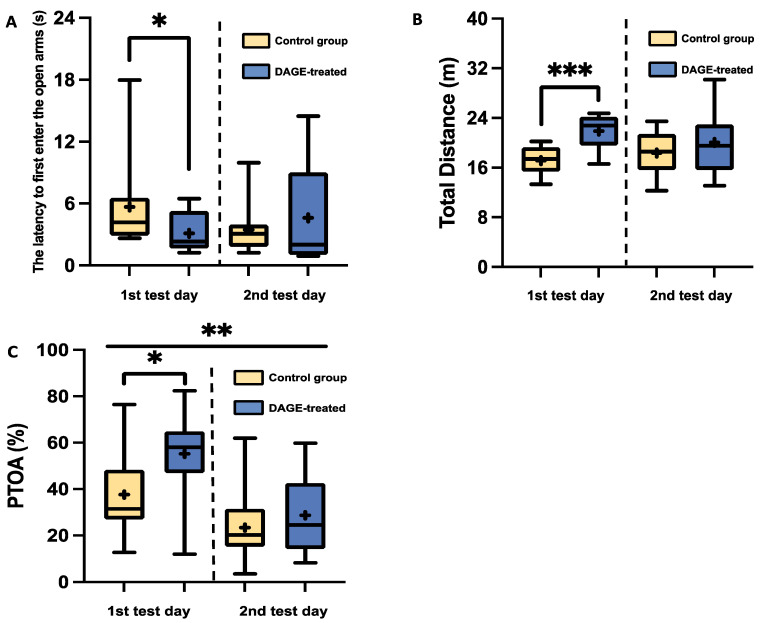
Box-and-Whisker Plots of Behavioral Metrics in C57BL/6 Mice: The plots illustrate the distribution of the following: (**A**) Latency to first entering the open arms; (**B**) Total distance traveled; (**C**) Percentage of time spent in open arms for DAGE-treated and control (non-DAGE-treated) groups. The boxes represent the 25th and 75th percentiles, while the whiskers extend to the 5th and 95th percentiles. The black lines within the boxes denote median values, and the “+” symbols indicate mean values. Asterisks indicate significance levels, with *, **, and *** representing *p*-values less than 0.05, 0.01, and 0.001, respectively.

In the C57BL/6 strain, DAGE’s effect on reducing anxiety is evident from the results shown in Figure 4A–C. On the first day of testing, DAGE-treated mice demonstrated a significantly shorter latency to first entering the open arms compared to the control mice, indicating reduced anxiety (*p* < 0.05), as depicted in Figure 4A. Moreover, these mice traveled a significantly greater distance throughout the test period than their control counterparts (*p* < 0.001), as shown in Figure 4B. Furthermore, Figure 4C shows that DAGE-treated mice spent a significantly greater percentage of time in the open arms on the first test day compared to controls (*p* < 0.05). Additionally, the data reveal a significant reduction in the percentage of time spent in the open arms for both treated and control groups on the second test day relative to the first (*p* < 0.01).

**Figure 5:**
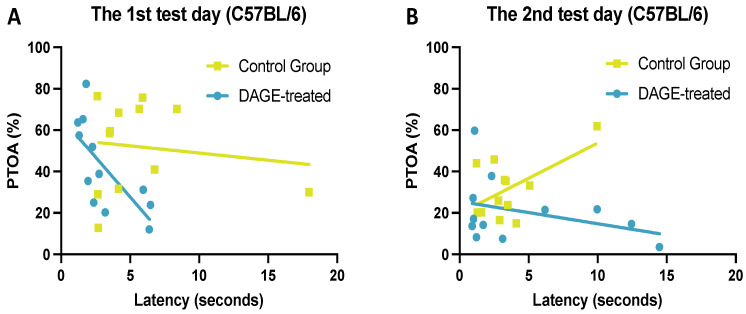
Correlation Between DAGE Treatment and Relief of Anxiety-Related Behavior: The latency to reaching the end of the open arms is used to assess exploratory behavior. The PTOA is used to denote an anxiety state. (**A**) Shows a linear relationship between PTOA and latency to the open-arm end in DAGE-treated mice (blue symbols and line) as well as between PTOA and latency to the open-arm end in control mice (yellow symbols and line), indicating a correlation between DAGE treatment and the relief of anxiety-related behavior on the first test day. (**B**) Demonstrates that on the second test day, this correlation was not observed (DAGE-treated, *R*^2^ = 0.119, *p* = 0.272; Control, *R*^2^ = 0.32, *p* = 0.06).

To further explore the impact of DAGE treatment on reducing anxiety-related behaviors, we analyzed the correlation between PTOA and latency to entering these arms across both groups of mice. Figure 5A reveals a significant negative correlation in DAGE-treated mice on the first test day, indicated by blue symbols (*R*^2^ = 0.501, *p* < 0.05), suggesting that increased time in open arms correlates with faster entry. Conversely, no significant correlation was found in the control group, as shown by the yellow symbols (*R*^2^ = 0.018, *p* = 0.674).

#### 3.1.2. Open-Field Test

**Figure 6:**
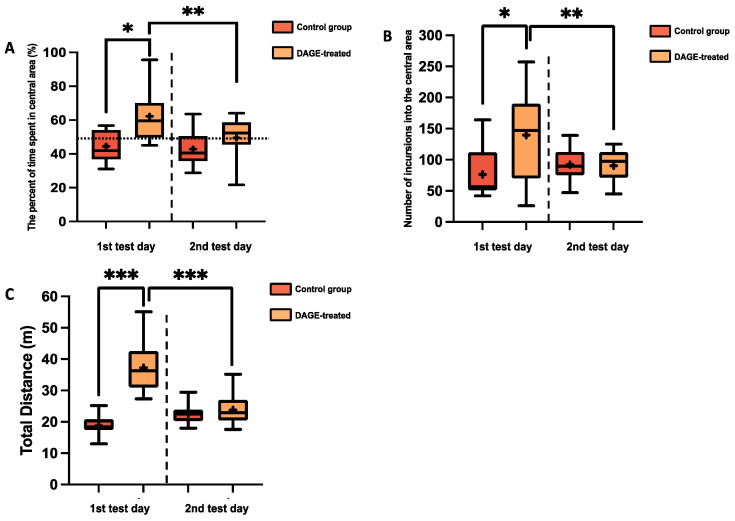
Box-and-Whisker Plots of Behavioral Metrics in BALB/c Mice: The plots illustrate the distribution of the following: (**A**) The percentage of time spent in the central area; (**B**) The number of incursions into the central area; (**C**) Total distance traveled through DAGE-treated and Control. The boxes represent the 25th and 75th percentiles, while the whiskers extend to the 5th and 95th percentiles. The black lines inside the boxes denote median values, and the “+” symbols indicate mean values. Asterisks indicate significance levels, with *, **, and *** representing *p*-values less than 0.05, 0.01, and 0.001, respectively.

The OFT was utilized to evaluate anxiety-related behaviors and exploratory activities in BALB/c mice, with results displayed in Figure 6A–C. On the first day of testing, DAGE-treated mice showed significant anti-anxiety effects, as evidenced by an increased percentage of time spent in the central area (*p* < 0.05; Figure 6A) and a higher number of entries into this area (*p* < 0.05; Figure 6B). These metrics are commonly employed to gauge anxiety levels in the OFT. Additionally, DAGE-treated mice covered a significantly greater total distance compared to controls (*p* < 0.001), indicating enhanced activity **(**Figure 6C). However, the anti-anxiety effects of DAGE treatment were notably diminished on the second test day (*p* < 0.01), with no significant differences observed compared to the control group.

**Figure 7:**
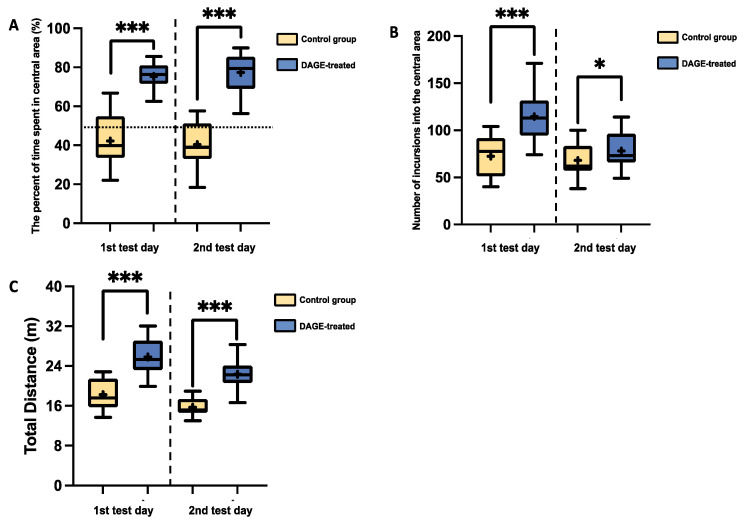
Box-and-Whisker Plots of Behavioral Metrics in C57BL/6 Mice: The plots illustrate the distribution of the following: (**A**) The percentage of time spent in the central area; (**B**) The number of incursions into the central area; (**C**) Total distance traveled through DAGE-treated and Control groups. The boxes represent the 25th and 75th percentiles, while the whiskers extend to the 5th and 95th percentiles. The black lines inside the boxes denote median values, and the “+” symbols indicate mean values. Asterisks indicate significance levels, with * and *** representing *p*-values less than 0.05 and 0.001, respectively.

In contrast, for the C57BL/6 strain, DAGE-treated mice exhibited significant anti-anxiety effects during the OFT. On the first test day, these mice spent a significantly longer percentage of time in the central area (*p* < 0.001; Figure 7A), entered the central area more frequently (*p* < 0.001; Figure 7B), and traveled a greater total distance compared to the control group (*p* < 0.001; Figure 7C). The results on the second test day also showed that DAGE-treated mice outperformed the control group in all assessed metrics: percentage of time spent in the central area (*p* < 0.001), number of entries into the central area (*p* < 0.05), and total distance traveled (*p* < 0.001). These findings underscore the sustained anti-anxiety effects of DAGE treatment in this mouse strain.

#### 3.1.3. Forced Swimming Test

**Figure 8:**
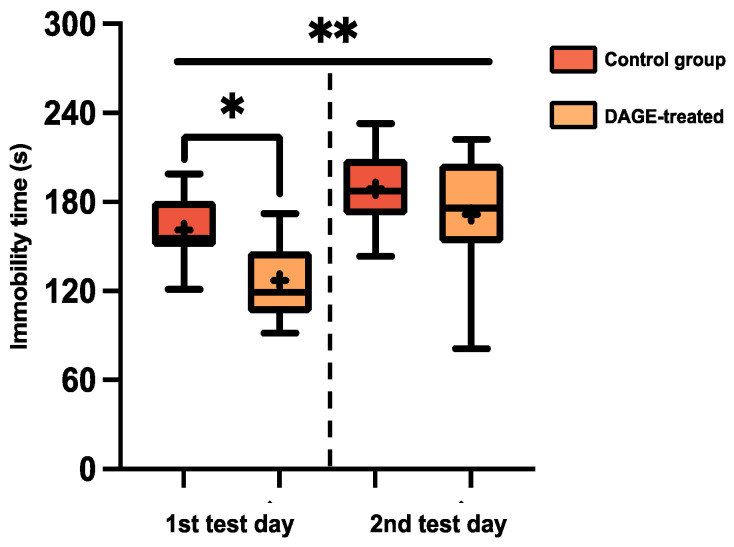
Box-and-Whisker Plots of Immobility Time for BALB/c Mice: These plots show the distribution of immobility times in BALB/c mice, comparing DAGE-treated and control groups. The boxes represent the 25th and 75th percentiles, while the whiskers extend to the 5th and 95th percentiles. Black lines inside the boxes denote median values, and “+” symbols indicate mean values. Asterisks represent statistical significance, with * and ** denoting *p*-values less than 0.05 and 0.01, respectively.

**Figure 9:**
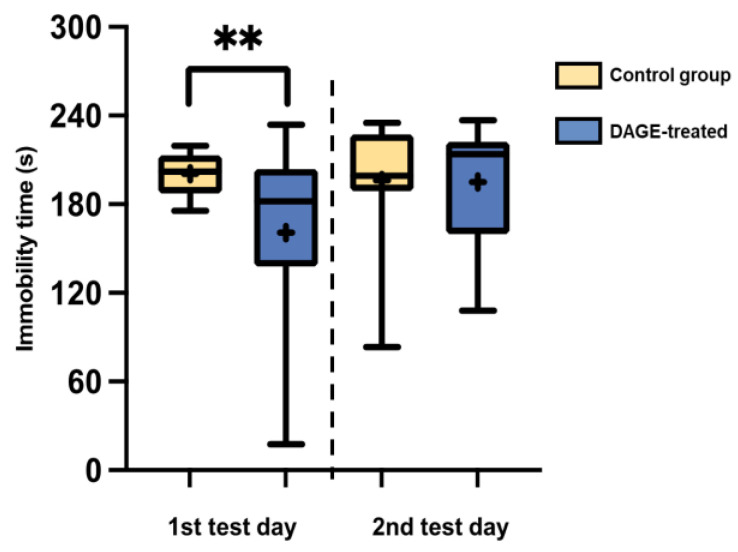
Box-and-Whisker Plots of Immobility Time for C57BL/6 Mice: These plots display the distribution of immobility times among C57BL/6 mice, comparing DAGE-treated mice to the control group. The boxes illustrate the 25th and 75th percentiles, while the whiskers extend to the 5th and 95th percentiles. Black lines inside the boxes represent median values, and “+” symbols indicate mean values. Double asterisks (**) denote statistical significance with a *p*-value less than 0.01.

The antidepressant effect of DAGE was evaluated using the FST, with results displayed in Figure 8 and Figure 9. Figure 8 illustrates the significant antidepressant effect of DAGE in BALB/c mice on the first test day, showing that chronic DAGE treatment significantly reduced immobility time (*p* < 0.05). However, on the second test day, immobility time significantly increased for all groups (*p* < 0.01).

Figure 9 illustrates the effects of DAGE treatment on C57BL/6 mice during the FST. On the first test day, DAGE-treated mice exhibited a significant reduction in immobility time compared to the control group (*p* < 0.01).

### 3.2. Changes in Body Weight per Food Intake

#### 3.2.1. Elevated Plus Maze

**Figure 10:**
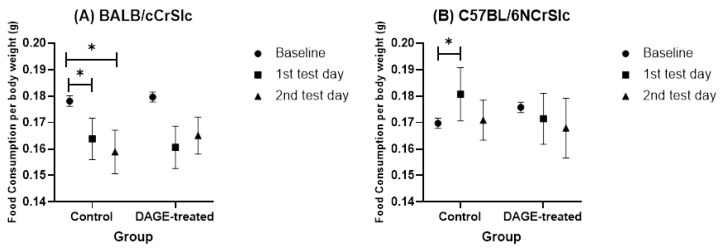
Impact of Long-term DAGE Consumption on Ingestive Behavior Following the EPM Test in Mice. This figure shows the changes in food consumption relative to body weight in mice before and after exposure to the EPM test. (**A**) Shows changes in food intake per unit of body weight in BALB/c mice. (**B**) Shows similar data for C57BL/6 mice. Icons represent different measurement points: the circle icon denotes the baseline mean value of food consumed before any behavioral tests were conducted. The square icon represents the average food intake during the 24 h period following the first EPM exposure. The triangle icon reflects the average intake within 24 h after the second EPM trial. * means *p* < 0.05.

Figure 10 illustrates the food consumption of each group during the 24 h following the EPM test. The data indicate that mice chronically treated with DAGE did not show significant changes in eating patterns, such as inhibited ingestion or binge eating behaviors after the EPM (*p* = ns). In contrast, the control group exhibited distinct behaviors: BALB/c mice demonstrated significantly reduced food intake after both test days (first test day: *t* = 2.773, *df* = 11, *p* < 0.05; second test day: *t* = 2.477, *df* = 11, *p* < 0.05), as shown in Figure 10A. C57BL/6 mice, displayed in Figure 10B, engaged in binge eating behaviors within the 24 h following the first test day (*t* = 2.336, *df* = 11, *p* < 0.05), indicating a significant increase in food intake.

#### 3.2.2. Open-Field Test

**Figure 11:**
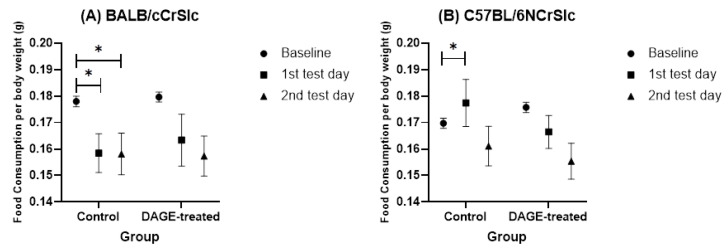
Impact of Long-term DAGE Consumption on Ingestive Behavior Following the OFT Test in Mice. This figure shows the changes in food consumption relative to body weight in mice before and after exposure to the OFT test. (**A**) Shows changes in food intake per unit of body weight in BALB/c mice. (**B**) Shows similar data for C57BL/6 mice. Icons represent different measurement points: the circle icon denotes the baseline mean value of food consumed before any behavioral tests were conducted. The square icon represents the average food intake during the 24 h period following the first OFT exposure. The triangle icon reflects the average intake within 24 h after the second OFT trial. * means *p* < 0.05.

Figure 11 presents the food consumption of each group within 24 h after participating in the OFT. The data indicate that mice receiving long-term DAGE treatment did not show significant changes in eating behaviors, such as inhibited ingestion or binge eating, after the OFT (*p* = ns). However, for the control group, BALB mice (Figure 11A) displayed significantly reduced food intake following both test days (first test day: *t* = 2.929, *df* = 11, *p* < 0.05; second test day: *t* = 2.799, *df* = 11, *p* < 0.05), indicating inhibited ingestion behaviors. In contrast, C57BL/6 mice (Figure 11B) exhibited binge eating behaviors within 24 h following the first test day (*t* = 2.497, *df* = 11, *p* < 0.05), indicating a significant increase in food consumption.

#### 3.2.3. Forced Swimming Test

**Figure 12:**
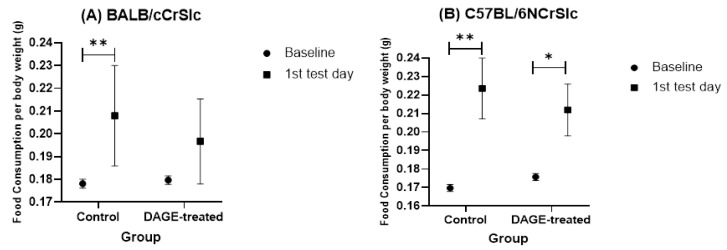
Impact of Long-term DAGE Consumption on Ingestive Behavior Following the FST. This figure shows food consumption relative to body weight in mice before and after the FST. (**A**) Shows the changes in food intake per unit of body weight in BALB/c mice before and after the FST. (**B**) Depicts similar data for C57BL/6 mice. The baseline (represented by a circle icon) indicates the mean value of food consumed before any behavioral tests were conducted. The square icon shows the average food intake during the 24 h following the initial FST exposure. It is important to note that after the second FST session, all mice were humanely euthanized, limiting data availability to the 24 h period following the first test only. * and ** mean *p* < 0.05 and 0.01.

Figure 12 details the food consumption of each group within 24 h after the FST. The data showed that BALB mice in the control group exhibited a significant increase in food intake after the FST (*t* = 2.872, *df* = 11, *p* < 0.01). In C57BL/6 mice, both control (*t* = 3.104, *df* = 11, *p* < 0.01) and DAGE-treated (*t* = 2.452, *df* = 11, *p* < 0.05) groups experienced significant increases in food intake within 24 h post-test

## 4. Discussion

In this study, we assessed the effects of DAGE treatment on anxiety- and depression-related behaviors in BALB/c and C57BL/6 mice using behavioral tests including the EPM, OFT, and FST. Our findings demonstrated that DAGE exerted pronounced anxiolytic effects in both strains, as shown by enhanced exploratory behaviors in the EPM and increased activity in the central areas of the OFT. Additionally, DAGE treatment resulted in reduced post-stress inhibited ingestion in BALB/c mice and decreased binge eating in C57BL/6 mice.

However, findings regarding the antidepressant effects of DAGE were less conclusive. Initial reductions in immobility time during the FST suggested possible antidepressant properties, but these were not consistently observed in the second test. This inconsistency implies that while DAGE treatment is effective in reducing anxiety, its effects on depressive behaviors are more variable and may depend on factors such as the mouse strain and specific stressors applied [38,39]. Consequently, while DAGE holds promise as an anxiolytic agent, further studies are needed to fully understand its potential in treating depressive symptoms.

The literature suggests that DAGE not only inhibit the reduction of leukocytes during radiotherapy but also enhance the production of immunoglobulins in saliva [10,40,41,42]. As a precursor to plasmalogens [10], DAGE facilitate the scavenging of reactive oxygen species through their vinyl ether bond, functioning as endogenous antioxidants [11]. Studies have shown that oxidative stress, particularly in sleep-deprived individuals, leads to decreased plasmalogen levels [12]. By consuming DAGE, the precursor to plasmalogens, one can increase plasmalogen levels, thereby mitigating brain oxidative stress. This reduction in stress is believed to enhance sleep quality and reduce anxiety [13]. Our findings align with this perspective, which demonstrate that DAGE supplementation not only increases activity levels but also the desire to explore in the Elevated Plus Maze and Open Field Tests. These increases in activity and exploration consequently lead to improvements in anxiety-related behaviors. Although previous research has pointed to antioxidant mechanisms as the basis for the anxiety-reducing properties of DAGE [11,13], this study did not directly measure oxidative stress markers. Future research should measure plasmalogen levels to validate the impact of DAGE on anxiety- and depression-related behaviors. Additionally, incorporating biochemical assays and brain imaging could provide deeper insights into the underlying biological mechanisms.

DAGE have demonstrated potential for alleviating symptoms related to anxiety and depression, with the most notable effects observed on the first test day. In our study, the mice receiving DAGE treatment exhibited significantly greater behavioral improvements in stressful and despairing environments than those given standard control food. Moreover, DAGE treatment enhanced overall activity levels, as evidenced by increased total distance traveled, and boosted exploratory behavior, as indicated by reduced latency to exploring. These findings position DAGE as a promising supplement for the prevention and mitigation of anxiety and depression [13]. However, while the initial data are encouraging, verifying these effects through clinical trials is crucial to confirm the efficacy of DAGE supplementation in a clinical setting.

Despite ongoing debates regarding the inherent anxiety levels of BALB/c and C57BL/6 mice [9,33,43,44,45], our study demonstrated that both strains, when treated with DAGE supplementation, showed significant reductions in anxiety and depressive behaviors on the initial test day compared to the controls. Notably, DAGE-treated BALB/c mice displayed variability in their OFT performance across the first and second days, suggesting that genetic factors may influence the effectiveness of DAGE treatment. This variability was also observed in the EPM in the C57BL/6 group, indicating potential genetic interactions that affect treatment outcomes [46]. Additionally, the control groups of both BALB/c and C57BL/6 mice exhibited distinct feeding responses to stress. While BALB/c mice reduced their food intake in response to stressors, including the EPM and OFT, C57BL/6 mice exhibited binge eating patterns under similar conditions. Following the FST, however, both strains increased their food intake, which is consistent with the findings of previous research [35,36]. These observations emphasize that different mouse strains may adopt varying adaptive strategies to cope with stress [38,39]. Future research should focus on identifying genetic determinants that influence responses to DAGE treatment, which could elucidate the mechanisms driving stress-related behaviors in diverse strains.

Behavioral differences observed across the two test days are typically attributed to various emotional responses. For instance, in the EPM, behaviors on the first test day are predominantly influenced by anxiety, whereas on the second test day, they are more driven by fear [16,22,47]. Similarly, in the FST, immobile behavior on the first day is often linked to depression-like emotions, while on the second day, it tends to be associated with unpleasant memories [48]. Given these findings, the less pronounced effects of DAGE treatment on the second test day may be partially due to shifts in emotional states; that is, while DAGE treatment is effective in alleviating anxiety and depression, its impact may diminish when these conditions transition to fear or unpleasant memories. However, limitations of our study, such as budget constraints that restricted access to necessary equipment, prevented us from confirming whether DAGE can cross the blood-brain barrier to directly influence brain states and functions, unlike fatty acids, such as DHA [49,50]. Further research is needed to determine the in vivo effects of DAGE and their interactions to fully understand the neural mechanisms underlying their effects on behavior.

In human studies, nutritional supplements containing a combination of DHA, EPA, and DAGE have been shown to enhance sleep quality [13], particularly by increasing the proportion of non-rapid eye movement (REM) sleep stage N3 and REM sleep stage R. These supplements also reduced daytime dysfunction and improved mood states, with participants reporting lower depression and tension scores and overall reduced mood disturbance. These benefits were particularly notable in individuals with low vigor and high stress levels, suggesting that the supplement offers significant anti-stress advantages [13]. However, it is important to note that these supplements also contained DHA and EPA, both of which have been previously documented to positively impact stress management [1,2]. Therefore, the specific contributions of DAGE to the observed improvements in sleep and anxiety cannot be conclusively isolated. While our results do support the potential effectiveness of DAGE supplementation in reducing anxiety and depression [13], our initial exploratory study did not include direct comparisons with traditional anxiolytic medications, such as benzodiazepines or SSRIs. Future research should aim to compare these supplements with conventional medications to more accurately delineate their mechanisms of action and relative efficacy.

The present study revealed a potential alleviating effect of DAGE on anxiety-related behaviors in mice. However, it is crucial to acknowledge certain limitations that highlight areas for further research. Firstly, the duration of the study was limited to a few weeks, which restricted our understanding of the long-term effects and sustainability of DAGE’s benefits. To address this, future studies should consider extended observation periods to more thoroughly investigate potential long-term side effects and changes in efficacy. Secondly, environmental factors such as housing conditions, which were not varied in this study, could significantly influence behavioral outcomes. Subsequent research should include these environmental variables (such as multi-cage environment and enriched environment) to determine their impact on the efficacy of DAGE and ensure that the results are robust across different settings. Finally, our study employed only male mice, which may limit the generalizability of the findings across genders. Including female mice in future research will not only help explore potential gender-related behavioral differences but also shed light on hormonal influences that could affect the efficacy of DAGE.

## 5. Conclusions

To our knowledge, this study is the first to investigate the anxiolytic and antidepressant effects of DAGE, a compound derived from deep-sea shark liver oil. The current results suggest that DAGE, derived from deep-sea shark liver oil, has potential anxiolytic and antidepressant effects. Our results indicate that DAGE-treated mice exhibited significant reductions in anxiety and depressive behaviors on the initial test day. These effects appear to be linked to DAGE’s role in mitigating oxidative stress within the brain. Our research enhances the understanding of the potential of DAGE treatment in stress management, highlighting how it interacts with various stress types, which could be relevant for human applications. Future research should focus on verifying the benefits of DAGE across repeated stress exposures and delve deeper into its active components and the underlying mechanisms of action. This will help solidify its use as a viable option for managing stress-related conditions in humans.

## Figures and Tables

**Table 1:** Composition of the DAGE and control foods.

	DAGE Food	Control Food
Composition	Basic formula: 94% (*w*/*w*) DAGE: 1.5% (*w*/*w*) Safflower oil: 4.5% (*w*/*w*)	Basic formula: 94% (*w*/*w*) Safflower oil: 6% (*w*/*w*)

Basic formula: AIN-93M soybean oil-free mature rodent feed (Research Diets, Inc. New Brunswick, NJ USA); DAGE, diacyl glyceryl ethers.

**Table 2:** Experimental group allocation.

Strain	Number of Mice	Treatment
BALB/cCrSIc	12	DAGE-enriched diet
BALB/cCrSIc	12	Control diet
C57BL/6NCrSIc	12	DAGE-enriched diet
BALB/cCrSIc	12	Control diet

DAGE, diacyl glyceryl ethers.

## Data Availability

The original contributions presented in the study are included in the article, further inquiries can be directed to the corresponding author.

## References

[1] Frautschy S.A., Cole G.M. (2011). What was lost in translation in the DHA trial is whom you should intend to treat. Alzheimer’s Res. Ther..

[2] Grosso G., Micek A., Marventano S., Castellano S., Mistretta A., Pajak A., Galvano F. (2016). Dietary n-3 PUFA, fish consumption and depression: A systematic review and meta-analysis of observational studies. J. Affect. Disord..

[3] Horikawa C., Otsuka R., Kato Y., Nishita Y., Tange C., Rogi T., Kawashima H., Shibata H., Ando F., Shimokata H. (2018). Longitudinal association between n-3 long-chain polyunsaturated fatty acid intake and depressive symptoms: A population-based cohort study in Japan. Nutrients.

[4] Hibbeln J.R., Nieminen L.R., Blasbalg T.L., Riggs J.A., Lands W.E. (2006). Healthy intakes of n−3 and n–6 fatty acids: Estimations considering worldwide diversity. Am. J. Clin. Nutr..

[5] Müller C.P., Reichel M., Mühle C., Rhein C., Gulbins E., Kornhuber J. (2015). Brain membrane lipids in major depression and anxiety disorders. Biochim. Biophys. Acta..

[6] Saini R.K., Keum Y.-S. (2018). Omega-3 and omega-6 polyunsaturated fatty acids: Dietary sources, metabolism, and significance—A review. Life Sci..

[7] Burokas A., Arboleya S., Moloney R.D., Peterson V.L., Murphy K., Clarke G., Stanton C., Dinan T.G., Cryan J.F. (2017). Targeting the microbiota-gut-brain axis: Prebiotics have anxiolytic and antidepressant-like effects and reverse the impact of chronic stress in mice. Biol. Psychiatry.

[8] Naeem S., Ali L., Rizwani G.H., Ikram R., Khan S.S., Shareef H., Younus I., Malick T.Z., Aleem U. (2020). A comparative neurobehavioral study of sesame oil and fish oil on experimental animals. Pak. J. Pharm. Sci..

[9] You R., Liu Y., Chang R.C.-C. (2019). A behavioral test battery for the repeated assessment of motor skills, mood, and cognition in mice. J. Vis. Exp..

[10] Destaillats F., Bezelgues J.-B., Dionisi F., Cruz-Hernandez C., Masserey-Elmelegy I. (2011). Method for Increasing Endogenous Plasmalogen Levels in Mammals. U.S. Patent.

[11] Engelmann B. (2004). Plasmalogens: Targets for oxidants and major lipophilic antioxidants. Biochem. Soc. Trans..

[12] Chua E.C.-P., Shui G., Cazenave-Gassiot A., Wenk M.R., Gooley J.J. (2015). Changes in plasma lipids during exposure to total sleep deprivation. Sleep.

[13] Hagu Y.A.N., Yuka T., Masaya K., Sumio K., Kousei N. (2021). Effect of a supplement product containing salmon roe oil-derived DHA and EPA and deep-sea shark liver oil-derived DAGE on the sleep quality and stress in healthy adults who have difficulties sleeping-a randomized, double-blind, placebo controlled, parallel group study. Jpn. Pharmacol. Ther..

[14] Pawlak C.R., Ho Y.-J., Schwarting R.K. (2008). Animal models of human psychopathology based on individual differences in novelty-seeking and anxiety. Neurosci. Biobehav. Rev..

[15] Komada M., Takao K., Miyakawa T. (2008). Elevated plus maze for mice. J. Vis. Exp..

[16] Pawlak C.R., Karrenbauer B.D., Schneider P., Ho Y.-J. (2012). The elevated plus-maze test: Differential psychopharmacology of anxiety-related behavior. Emot. Rev..

[17] Gould T.D., Dao D.T., Kovacsics C.E., Gould T.D. (2009). The open field test. Mood and Anxiety Related Phenotypes in Mice: Characterization Using Behavioral Tests.

[18] Seibenhener M.L., Wooten M.C. (2015). Use of the open field maze to measure locomotor and anxiety-like behavior in mice. J. Vis. Exp..

[19] Can A., Dao D.T., Arad M., Terrillion C.E., Piantadosi S.C., Gould T.D. (2012). The mouse forced swim test. J. Vis. Exp..

[20] Porsolt R.D., Le Pichon M., Jalfre M. (1977). Depression: A new animal model sensitive to antidepressant treatments. Nature.

[21] Tadano T., Abe Y., Morikawa Y., Hozumi M., Nakagawasai O., Tan-No K., Kisara K. (1997). Relationship between learning behavior and genetic factor on immobility shown during forced swimming test. Nihon Shinkei Seishin Yakurigaku Zasshi.

[22] Albrechet-Souza L., Borelli K.G., Brandao M.L. (2008). Activity of the medial prefrontal cortex and amygdala underlies one-trial tolerance of rats in the elevated plus-maze. J. Neurosci. Methods.

[23] Frussa-Filho R., Ribeiro R.D.A. (2002). One-trial tolerance to the effects of chlordiazepoxide in the elevated plus-maze is not due to acquisition of a phobic avoidance of open arms during initial exposure. Life Sci..

[24] Bolivar V.J., Caldarone B.J., Reilly A.A., Flaherty L. (2000). Habituation of activity in an open field: A survey of inbred strains and F1 hybrids. Behav. Genet..

[25] Gil M., Armario A. (1998). Chronic immobilization stress appears to increase the role of dopamine in the control of active behaviour in the forced swimming test. Behav. Brain. Res..

[26] Gershenfeld H.K., Neumann P.E., Mathis C., Crawley J.N., Li X., Paul S.M. (1997). Mapping quantitative trait loci for open-field behavior in mice. Behav. Genet..

[27] Cabib S., Puglisi-Allegra S. (1996). Stress, depression and the mesolimbic dopamine system. Psychopharmacology.

[28] File S.E., Mabbutt P.S., Hitchcott P.K. (1990). Characterisation of the phenomenon of “one-trial tolerance” to the anxiolytic effect of chlordiazepoxide in the elevated plus-maze. Psychopharmacology.

[29] Garcia-Marquez C., Armario A. (1987). Chronic stress depresses exploratory activity and behavioral performance in the forced swimming test without altering ACTH response to a novel acute stressor. Physiol. Behav..

[30] Carola V., D’Olimpio F., Brunamonti E., Bevilacqua A., Renzi P., Mangia F. (2004). Anxiety-related behaviour in C57BL/6↔BALB/c chimeric mice. Behav. Brain. Res..

[31] Pellow S., Chopin P., File S.E., Briley M. (1985). Validation of open: Closed arm entries in an elevated plus-maze as a measure of anxiety in the rat. J. Neurosci. Methods.

[32] Walf A.A., Frye C.A. (2007). The use of the elevated plus maze as an assay of anxiety-related behavior in rodents. Nat. Protoc..

[33] Trullas R., Skolnick P. (1993). Differences in fear motivated behaviors among inbred mouse strains. Psychopharmacology.

[34] Bello-Arroyo E., Roque H., Marcos A., Orihuel J., Higuera-Matas A., Desco M., Caiolfa V.R., Ambrosio E., Lara-Pezzi E., Gómez-Gaviro M.V. (2018). MouBeAT: A new and open toolbox for guided analysis of behavioral tests in mice. Front. Behav. Neurosci..

[35] Razzoli M., Carboni L., Andreoli M., Ballottari A., Arban R. (2011). Different susceptibility to social defeat stress of BalbC and C57BL6/J mice. Behav. Brain. Res..

[36] Consoli D., Contarino A., Tabarin A., Drago F. (2009). Binge-like eating in mice. Int. J. Eat. Disord..

[37] Groesz L.M., McCoy S., Carl J., Saslow L., Stewart J., Adler N., Laraia B., Epel E. (2012). What is eating you? Stress and the drive to eat. Appetite.

[38] Sugimoto Y., Kajiwara Y., Hirano K., Yamada S., Tagawa N., Kobayashi Y., Hotta Y., Yamada J. (2008). Mouse strain differences in immobility and sensitivity to fluvoxamine and desipramine in the forced swimming test: Analysis of serotonin and noradrenaline transporter binding. Eur. J. Pharmacol..

[39] David D.J.P., Renard C.E., Jolliet P., Hascoët M., Bourin M. (2003). Antidepressant-like effects in various mice strains in the forced swimming test. Psychopharmacology.

[40] Hamadate N., Shikura M., Mizukami C., Seto K., Yamamoto T., Yamagichi H., Izuka M., Yamamoto E., Kondo S., Yazawa K. (2015). Effect of the deep sea shark-liver oil component food on secretion type immunoglobulin a density of saliva in the normal man and women adult. Jpn. Soc. Complement. Altern. Med..

[41] Das A.K., Hajra A.K. (1988). High incorporation of dietary 1-O-heptadecyl glycerol into tissue plasmalogens of young rats. FEBS Lett..

[42] Brohult A., Brohult J., Brohult S., Joelsson I. (1977). Effect of alkoxyglycerols on the frequency of injuries following radiation therapy for carcinoma of the uterine cervix. Acta. Obstet. Gynecol. Scand..

[43] Depino A.M., Gross C. (2007). Simultaneous assessment of autonomic function and anxiety-related behavior in BALB/c and C57BL/6 mice. Behav. Brain. Res..

[44] Carola V., D’Olimpio F., Brunamonti E., Mangia F., Renzi P. (2002). Evaluation of the elevated plus-maze and open-field tests for the assessment of anxiety-related behaviour in inbred mice. Behav. Brain. Res..

[45] Rogers D.C., Jones D.N., Nelson P.R., Jones C.M., Quilter C.A., Robinson T.L., Hagan J.J. (1999). Use of SHIRPA and discriminant analysis to characterise marked differences in the behavioural phenotype of six inbred mouse strains. Behav. Brain. Res..

[46] Anderzhanova E.A., Bächli H., Buneeva O.A., Narkevich V.B., Medvedev A.E., Thoeringer C.K., Wotjak C.T., Kudrin V.A. (2013). Strain differences in profiles of dopaminergic neurotransmission in the prefrontal cortex of the BALB/C vs. C57Bl/6 mice: Consequences of stress and afobazole. Eur. J. Pharmacol..

[47] File S.E. (1993). The interplay of learning and anxiety in the elevated plus-maze. Behav. Brain. Res..

[48] Su J., Hato-Yamada N., Araki H., Yoshimura H. (2013). A test–retest paradigm of the forced swimming test in female mice is not valid for predicting antidepressant-like activity: Participation of acetylcholine and Sigma-1 receptors. J. Pharmacol. Sci..

[49] Sugasini D., Thomas R., Yalagala P.C., Tai L.M., Subbaiah P.V. (2017). Dietary docosahexaenoic acid (DHA) as lysophosphatidylcholine, but not as free acid, enriches brain DHA and improves memory in adult mice. Sci. Rep..

[50] Nguyen L.N., Ma D., Shui G., Wong P., Cazenave-Gassiot A., Zhang X., Wenk M.R., Goh E.L.K., Silver D.L. (2014). Mfsd2a is a transporter for the essential omega-3 fatty acid docosahexaenoic acid. Nature.

